# From infection to cancer: how DNA tumour viruses alter host cell central carbon and lipid metabolism

**DOI:** 10.1098/rsob.210004

**Published:** 2021-03-03

**Authors:** Kamini L. Magon, Joanna L. Parish

**Affiliations:** Institute of Cancer and Genomic Science, College of Medical and Dental Sciences, University of Birmingham, Birmingham B15 2TT, UK

**Keywords:** oncogenic DNA viruses, virus–host interactions, metabolism, lipid, central carbon

## Abstract

Infections cause 13% of all cancers globally, and DNA tumour viruses account for almost 60% of these cancers. All viruses are obligate intracellular parasites and hijack host cell functions to replicate and complete their life cycles to produce progeny virions. While many aspects of viral manipulation of host cells have been studied, how DNA tumour viruses manipulate host cell metabolism and whether metabolic alterations in the virus life cycle contribute to carcinogenesis are not well understood. In this review, we compare the differences in central carbon and fatty acid metabolism in host cells following infection, oncogenic transformation, and virus-driven cancer of DNA tumour viruses including: Epstein–Barr virus, hepatitis B virus, human papillomavirus, Kaposi's sarcoma-associated herpesvirus and Merkel cell polyomavirus.

## Graphical abstract

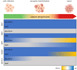

## 1. Introduction

All viruses, including DNA tumour viruses, depend on host cell function for life cycle completion, including the hijacking of host metabolic processes and the use of host macromolecules. Of the 13% of cancers which are caused by infections globally, oncogenic DNA tumour viruses account for almost 60% of these cancers [1], and therefore represent a significant health burden. It was determined in the 1950s by Otto Warburg that tumours have a different metabolic profile to normal tissues [2] and since then, there has been an increase in research focused on tumour metabolism, with altered cellular metabolism defined as a hallmark of cancer [3]. However, it remains unclear whether viral alterations to metabolism contribute to carcinogenesis, and if the changes observed in productive infections contribute to cancer development and are maintained in the tumour. This review summarizes the changes in host metabolism during viral infection and oncogenic DNA tumour virus-driven cancers.

## 2. Epstein–Barr virus

Epstein–Barr virus (EBV) is a gamma herpesvirus that is highly prevalent in the human population; nearly all adults are seropositive. EBV can infect both B lymphocytes and epithelial cells, and as a result causes a variety of clinical outcomes, including infectious mononucleosis [4]. Persistent EBV infection drives a variety of lymphomas, including 30–62% of Hodgkin's lymphomas, 20–95% of Burkitt lymphomas and 80% of post-transplant lymphoproliferative disease, as well as epithelial cancers, including 80–100% of nasopharyngeal cancers (NPC) and 6–11% of gastric cancers [5]. EBV is a well-studied virus, and there has been a significant interest in the ways it alters host glucose and lipid metabolism, in both infection and EBV-driven cancer.

### 2.1. Central carbon metabolism

The effects of EBV infection on B cell glucose metabolism have been investigated for many years, and it has been established that the overall metabolic profile of EBV-infected B cells is altered 3 days post infection. In agreement with these findings, glucose uptake increases following EBV infection within 4 days [6]. In proliferation-arrested EBV-infected primary B cells, decreased expression of genes involved in the tricarboxylic acid (TCA) cycle and oxidative phosphorylation compared to other B cell populations was observed, which could lead to the promotion of autophagy and senescence [7]. Furthermore, there was an increase in both glycolysis and oxidative phosphorylation as cells changed from hyperproliferative to immortal lymphoblastoid lines [7]. In EBV-transformed lymphoblastoid cells, hypoxia-inducible factor (HIF) 1*α* expression is increased and constitutively active, which increases the expression of HIF1α-responsive genes involved in aerobic glycolysis to promote the Warburg effect [8].

Similar findings have been identified in epithelial cells following EBV infection. In EBV-infected nasopharyngeal epithelial cells, expression of latent membrane protein 1 (LMP1) causes a change in the metabolic phenotype of the cells to favour glycolysis [9]. In agreement with this, overexpression of LMP1 in nasopharyngeal epithelial cells increased lactate production and glucose consumption, and resulted in an increase in the expression of several proteins including pyruvate kinase M2 (PKM2) and lactate dehydrogenase A1 (LDHA1) involved in glycolysis [10]. LMP1 expression also activates fibroblast growth factor receptor (FGFR) 1 signalling to mediate aerobic glycolysis, which promotes cell migration and invasion [10]. Indeed, when glycolysis was inhibited in EBV-infected cells, epithelial-to-mesenchymal transition (EMT), a process associated with oncogenic transformation of the cells, was decreased [9].

The metabolic alterations caused by EBV infection are similar to those identified in EBV-driven cancers, and viral LMP1 has a clear role in driving the metabolic changes observed in EBV-driven malignancies of both epithelial cell and B cell origin. Several studies have shown that expression of LMP1 induced aerobic glycolysis in NPC, in cell culture and patient-derived samples [11–15] by manipulating several different host cell pathways. For example, LMP1 increased the expression of hypoxia-inducible factor 1 alpha (HIF1*α*), which functions to increase the expression of genes involved in glycolysis; this was coupled with a decrease in oxygen consumption rate (OCR), suggesting a decrease in oxidative phosphorylation [11]. LMP1 expression in EBV-negative NPC cell lines increases expression of hexokinase (HK) 2, a rate limiting enzyme of glycolysis, which resulted in an increase in lactate production and glucose consumption, both of which are associated with an increase in aerobic glycolysis [12]. LMP1 also induced a decrease in expression of the homeobox gene C8 (HoxC8). Since overexpression of HoxC8 resulted in decreased glucose consumption and lactic acid production alongside decreased expression of hexokinase 2 (HK2) and glucose transporter (GLUT) 1 [13], this suggests that LMP1 may drive an aerobic glycolytic phenotype by decreasing HoxC8 expression [13]. In EBV-positive NPC cells, LMP1 drives DNA (cytosine-5)-methyltransferase (DNMT) 1 localization to mitochondria where it functions to inhibit oxidative phosphorylation and promote aerobic glycolysis [14]. Just as EBV viral proteins can interact with host cell factors to alter glycolysis, EBV-encoded viral miRNAs have been shown to alter host metabolism. Expression of a viral micro RNA (miRNA), BART1-5P, in NPC cells increased glucose consumption and lactate production by regulating the AMP-activated protein kinase (AMPK)/mTOR/HIF1*α* pathway [16]. While it is important to consider the impact of viral infection on host cell processes, within a tumour there are multiple additional cell types within the microenvironment which may be altered due to virus-driven cancers. Using a Transwell co-culture system, LMP1-expressing NPC cells were shown to promote the differentiation of myeloid-derived suppressor cells (MDSCs), a key component of the tumour microenvironment, and the expression of MDSC-related molecules including cytokines. The inhibition of GLUT1 in LMP1-expressing NPC cells decreased the expression of MDSC differentiation-related genes [15], suggesting that alterations to host cell metabolism induced by viral infection also impacts the microenvironment.

Most of the work describing EBV-mediated alteration of central carbon metabolism has been carried out in epithelial malignancies, and the understanding of EBV-driven B cell malignancies is limited in comparison. However, it has been demonstrated that EBV LMP1 protein expression decreases mitochondrial respiration and promotes aerobic glycolysis in a Burkitt lymphoma cell line [17]. These findings are in line with those seen in epithelial malignancies, suggesting that EBV drives and promotes aerobic glycolysis over oxidative phosphorylation to rapidly provide energy to the cells. It is clear that EBV manipulates host cell glucose metabolism to favour glycolysis, and this is likely to promote oncogenic progression and transformation. This glycolytic phenotype persists during malignancy in order to provide a survival advantage to tumour cells. One limitation in the current work is that there is a large focus on LMP1 in isolation and the field could benefit from investigating the impact of other EBV viral proteins, miRNAs and EBV replication on cellular glycolysis.

### 2.2. Lipid metabolism

EBV has been reported to manipulate host cell lipid metabolism in both epithelial and B cells. Expression of the immediate-early EBV protein BRLF1 induces expression of fatty acid synthase (FASN), which encodes an enzyme involved in fatty acid synthesis through activation of p38 mitogen-activated protein (MAP) kinase in telomerase-immortalized keratinocytes [18]. In newly infected B cells, there is a significant enrichment of proteins involved in fatty acid metabolism and cholesterol biosynthesis 4 days post infection, as well as the upregulation of growth-promoting transcription factors Myc and sterol regulatory element-binding protein (SREBP) 2. Treatment with simvastatin, a cholesterol-lowering agent, significantly reduces the number of EBV-infected B cells, highlighting the role cholesterol metabolism may play in the EBV life cycle [19].

Similar changes in fatty acid metabolism have also been identified in EBV-driven cancers. In EBV-associated gastric carcinoma, EBV significantly decreases the transcription of metabolic-associated genes as well as 69 out of 114 identified lipid species [20]. Inhibition of FASN in either cell lines derived from Burkitt lymphoma, which express BRLF1, or in EBV-positive gastric carcinoma cell lines resulted in a decrease in the expression of BRLF1-mediated lytic viral genes [18], suggesting the importance of fatty acid synthesis to EBV gene expression. Similarly, in LMP1-expressing nasopharyngeal epithelial cells, increased expression of SREBP1 mediates lipogenesis through mammalian target of rapamycin (mTOR) signalling; the induction of lipid synthesis promotes cell growth in EBV-driven cancers, and when inhibited, there is a suppression of growth and an increase in apoptosis in NPC cells [21]. EBV-encoded RNAs (EBERs) in NPC cells have also been shown to deregulate cellular lipid metabolism in a similar manner to EBV-associated proteins; 19 out of 54 genes upregulated by EBERs were shown to be involved in lipid metabolism, including SREBF1/2, FASN and low density lipoprotein receptor (LDLR), and inhibition of FASN significantly decreased cell growth [22]. EBV-driven manipulation of lipid metabolism is evident in both B cell and epithelial cell infection and malignancy, highlighted by an upregulation of synthesis pathways both in early infection and in cancers suggesting that manipulation of lipid metabolism may play a role in host cell transformation and carcinogenesis. Further utilization of cellular models of EBV-driven transformation will enable us to understand whether manipulation of host lipid pathways is a key driver of EBV-associated cancers.

## 3. Hepatitis B

Hepatitis B virus (HBV) is a cause of chronic liver disease and one of two viral aetiological agents of hepatocellular carcinoma (HCC). Globally, there are over 1.5 billion patients suffering with chronic liver disease, of which HBV infection contributes to 29% of these cases [23]. While there are multiple causes of HCC, including liver cirrhosis and heavy alcohol intake, HBV accounts for 44–55% of HCC [24]. The liver is a highly metabolically active organ and it is therefore unsurprising that HBV infection manipulates the metabolism of liver cells, particularly lipid metabolism pathways, in order to facilitate persistent infection and drive cancer development.

### 3.1. Central carbon metabolism

Our understanding of how HBV alters host glucose metabolism is currently limited, despite the active metabolic role of the liver. In a primary rat hepatocyte model, RNA sequencing and pathway analysis revealed a significant alteration to genes involved in the glycolysis pathway 72 h post HBV infection [25]. Similarly analysis of urine and serum from patients chronically infected with HBV identified several enzymes involved in the TCA cycle that were significantly downregulated [26], although this may not be via direct manipulation of host cell metabolism by HBV. The hepatoma-derived cell line, HepG2.2.15 which contain integrated copies of the HBV genome, maintain episomal covalently closed circular HBV DNA, and can produce infectious virus [27] show an altered metabolic profile compared to parental HepG2 cells. This includes an increase in both glucose utilization in the expression of many enzymes involved in glycolysis [28]. Similarly, expression of the multifunctional HBV protein, HBx, important in the maintenance of viral replication [29] and the pathogenesis of HCC [30], results in disrupted glucose metabolism as shown by a decrease in glucose and glucose-6-phosphate in HepG2 cells [31]. Furthermore, HBx transgenic mice displayed increased expression of gluconeogenesis-associated genes and impaired glucose metabolism, resulting in hyperglycaemia [32]. By contrast, HepG2 cells expressing only HBV core protein (HBc) displayed increased expression of proteins involved in glucose metabolism; this was similar to HBV infection of HepG2 cells. However, expression of only HBx in these cells decreases glucose expression [33], suggesting the different viral proteins have different effects on host cell glucose metabolism. The HBV pre-S2 mutant increases glucose uptake and lactate production, which are indicative of increased aerobic glycolysis through the mTOR/yin yang 1 (YY1)/Myc/solute carrier (SLC) 2A1 signalling cascade. The cascade is also induced in HBV-associated HCC by the pre-S2 mutant [34]. It has been reported that HepG2.2.15 cells grown in higher concentrations of glucose have lower copies of intracellular and secreted HBV DNA, highlighting that manipulation of glucose metabolism is critical for HBV survival and replication within host cells [35]. Overall, it remains unclear as to whether HBV infection drives a glycolytic phenotype following infection, but it is clear that glucose metabolism is altered, which may promote transformation. However, more work is needed to determine whether there are metabolic differences between HBV-induced HCC and HCC caused by other factors, in order to fully understand whether the changes persist and drive the proliferation of cancer cells.

### 3.2. Lipid metabolism

While the role of HBV in altering glucose metabolism has limited understanding, there is greater understanding of the ways in which HBV alters host lipid metabolism. cDNA microarray analysis of an HBV transgenic mouse system identified an upregulation of genes required for lipid and steroid biosynthesis and metabolism, including FASN and acetoacetyl-CoA synthetase (AACS) in liver tissue [36]. In HBc-expressing HepG2 cells, there is a significant increase in genes involved in fatty acid metabolism compared to the control cells [33]. Similarly, in HBV-infected HepG2.2.15 cells, there was a significant increase in the expression of phosphatidylcholine (PC), which was shown to be important for HBV replication [28]. Transfection of HepG2 and Huh7 cells with the full HBV genome resulted in an increase in lipid accumulation in these cells [37]. Some of the potential mechanisms through which HBV alters lipid metabolism have been investigated. Activation of the retinoid X receptor (RXR) inhibited HBV infection and arachidonic acid biosynthesis, which is formed from fatty acids, and its downstream metabolic pathways [38]. Similarly, HBV-transfected HCC cells showed an increase in lipogenic transcription factors including peroxisome proliferator activated receptor gamma (PPAR*γ*) and CCAAT-enhancer-binding protein alpha (C/EBP*α*) and induction of the expression of enzymes involved in saturated fatty acid synthesis, driven by mitochondrial dysfunction [37]. HBx overexpression has been shown to induce lipid accumulation in HepG2 cells and HBx-transgenic mice through inducing the expression of lipogenic and adipogenic genes through SREBP1 and PPAR*γ* [39]. Similarly, HBx has been shown to induce lipid accumulation in HepG2 cells and HBx transgenic mice by facilitating binding of hepatocyte nuclear factor (HNF) 3*β*, C/EBP*α*, and PPAR*α* to the fatty acid binding protein (FABP) 1 promoter to increase FABP1 expression, a key regulator in hepatic lipid metabolism [40]. Transfection of Chang cells (a HeLa cell line originally believed to be a human normal hepatocyte line [41]) with HBx resulted in enhanced expression of *N*-acetylglucosaminyltransferase-III (GnT-III), which resulted in an increase in aberrantly glycosylated apolipoprotein B (apoB). As a result of this, apoB is not able to be secreted, which results in an increase of intracellular triglyceride and cholesterol [42]. Mutant variants of the viral protein HBx are commonly found in patients chronically infected with HBV, and mice with mutations in HBx had a higher tumour burden than in mice with wild-type (WT) HBx. Furthermore, RNA-Seq and metabolomics analysis revealed that metabolic disorder was associated with tumour development, specifically, a decrease in the metabolites involved in the synthesis of bile acids from cholesterol, and arachidonic acid metabolic pathways were activated in mutant HBx genotypes [43]. Overall, it appears that HBV may increase lipid accumulation and synthesis suggesting that the alterations to aspects of host lipid metabolism facilitate persistent infection and may help to drive carcinogenesis.

Changes in lipid metabolism have also been identified in HBV-driven HCC. Analysis of the serum profiles of patients with chronic HBV (CHB), HBV-associated liver cirrhosis (LC), and HBV-driven HCC revealed that several lysophosphatidylcholine (lysoPC) species decreased between CHB, LC and HCC in a step-wise manner [44], suggesting that this may be relevant as a marker for disease progression and tumorigenesis. In HBV-associated HCC samples, there was significant downregulation of long-chain fatty acid metabolism and fatty acid degradation compared to adjacent healthy tissue [45]. Additionally, dysregulated triglyceride and cholesterol expression in HBx transgenic mice has been noted, as well as an increase in total saturated and monounsaturated fatty acids in liver tumours of mice. Furthermore, there was an activation of genes involved in multiple lipid metabolism-associated pathways at three months of age [46]. In an HBx-induced liver carcinogenesis mouse model, carcinogenesis is driven by deregulating lipid metabolism through mitochondrial dysfunction, and also increases the cholesterol and lipid content of cells [47]. In pre-S2 mutant transgenic mice which develop HCC, there was a higher level of lipid accumulation in HCC tissues through activation of ATP citrate lyase (ACLY) through mTOR/SREBF1 which promotes lipogenesis; the ACLY/mTOR/SREBF1 cascade is also activated in human HBV-associated HCC [48]. Overall, is it clear that HBV alters lipid metabolism throughout infection and carcinogenesis, particularly through an accumulation of lipids within hepatocytes. However, the understanding of HBV-driven transformation is somewhat limited and it is therefore difficult to determine whether the alterations seen in infection models promote oncogenic progression. There is a paucity of models for HBV-driven transformation that can be used to determine whether the changes seen in early and chronic infection are drivers of carcinogenesis.

## 4. High-risk human papillomaviruses

Human papillomavirus (HPV) infection affects nearly all sexually active adults [49], and can be subdivided into either low-risk (LR) types which cause warts, or high-risk (HR) types, which upon persistent infection cause nearly 5% of cancers worldwide [50]. HPVs infect basal keratinocytes of both mucosal and cutaneous epithelia, and as a result, HPV-driven cancers occur at a variety of anatomical sites, including the oropharyngeal and ano-genital regions.

### 4.1. Central carbon metabolism

The analysis of how HR-HPVs manipulate host metabolism is largely limited to the effects of single viral proteins. In a high glycolytic strain of 3T3 fibroblasts transformed with HPV16 E7 protein, there is a shift from the tetrameric form of PKM2, the key form in catalysing the last step in glycolysis, to the dimeric form, which has lower substrate activity and has been shown to accumulate in tumours [51]. HPV16 E7 binds strongly to PKM2, while HPV11 E7 (a LR-HPV) does not bind to PKM2. Furthermore, rat kidney cells transformed with HPV16 E7 [52] have a higher glycolytic rate and an increased conversion rate of glucose to lactate [53]. These findings suggest that oncogenic HR-HPV E7 may alter steps in glycolysis to promote survival during oncogenic transformation. However, it would be beneficial to repeat this work in primary human keratinocytes to gain a greater understanding of viral manipulation in a physiologically relevant model. The HPV E2 protein has also been implicated in altering host metabolism. HPV18 E2 expression in HaCaT cells increases reactive oxygen species production and HIF1*α* expression, which both play a role in the cellular response to hypoxia. As a consequence of increased HIF1*α* expression, there is an increase in transcription of its target genes, including pyruvate dehydrogenase kinase (PDK1) and carbonic anhydrase 9 (CAIX) [54]; PDK1 inhibits the pyruvate dehydrogenase (PDH) enzyme that converts pyruvate into acetyl-CoA, in effect inhibiting oxidative phosphorylation, and CAIX de-acidifies cells in response to lactate, which is produced from aerobic and anaerobic glycolysis. This suggests that E2 can prime cells to favour glycolysis over other methods of energy metabolism, and overall suggest that multiple proteins from HR-HPVs manipulate host metabolism. However, more work is needed further elucidate the role of metabolic manipulation of the host cell has in the viral life cycle, persistent infection and carcinogenesis.

HR-HPVs are the aetiological agents of nearly all cervical cancers which have well-defined stages of disease progression. Persistent infection of HR-HPV can result in the formation of cervical intraepithelial neoplasia (CIN), which is defined as a premalignant cervical lesion. CIN is divided into three grades of increasing clinical severity (CIN1-3), which can progress to carcinoma *in situ* [55]. This provides a useful tool for not only the screening and diagnosis of cervical cancer, but also for the investigation of multi-step carcinogenesis. In a study comparing different stages of CIN and cervical cancer to normal cervical tissue, it was identified that genes encoding proteins involved in the mitochondrial electron transport chain are downregulated in cervical cancer specimens compared to CIN3 [56], suggesting that cervical cancer may favour other pathways for energy production. However, while all of the samples in this study were confirmed positive for HPV infection, the healthy control tissues were also positive for HPV, and therefore the direct role of HPV in these changes cannot be determined. However, it is interesting to note that in both infection and cancer development, similar changes in glycolysis are observed.

While there is limited understanding of the effects of HPV on glycolysis manipulation in early infection, there is better understanding in HPV-driven cancer. Analysis of data from The Cancer Genome Atlas (TCGA) [57], patient samples and cell lines [58] revealed that HPV-positive head and neck cancers have a lower expression of genes involved in glycolysis, and may preferentially use oxidative phosphorylation, compared to HPV-negative tumours. Similarly, in oropharyngeal carcinoma samples, there was a higher expression of GLUT1, LDHB and monocarboxylate transporter (MCT) 1, three genes involved in glycolytic and oxidative metabolism, which was mirrored in the HPV-positive head and neck squamous cell carcinoma cell line, UD-SCC-2, further supporting the idea that HPV-driven cancer uses oxidative phosphorylation and the TCA cycle, over glycolysis [59]. The role of viral proteins in these alterations has been investigated. In the HPV16-positive SiHa and CaSki cell lines, the addition of recombinant viral L2 protein resulted in a significant decrease in glucose uptake and lactate production, and a lower level of maximal oxygen consumption rate, a measure of mitochondrial respiration, which together are indicative of reduced glycolysis [60]. This is thought to be due to L2 decreasing expression of genes involved in the integrin beta-7 (ITGB7)/ C/EBP*β* signalling pathway, as conversely overexpression of ITGB7 resulted in an increase in glucose and lactate production [60]. However, a decrease in glycolysis in HPV infected cells is not always observed. In HPV16-positive SiHa and HPV18-positive HeLa cells, it was reported that the viral E6 oncoprotein promotes glycolysis through decreasing the expression of miRNA-34a; knockdown of E6 increases miRNA-34a and subsequently decreases lactate and glucose consumption through a decrease in LDHA [61]. Similarly, in CaSki cells, HPV16-E6 knockdown results in a decrease in lactate and glucose production under hypoxic conditions; E6 was shown to inhibit the association between Von Hippel-Lindau (VHL) and HIF1*α*, suggesting that E6 expression promotes glycolysis under hypoxia by allowing HIF1*α* to accumulate [62]. While manipulation of individual viral proteins in cancer cell lines can aid our understanding of the roles they play in altering glycolysis, it is important to study these alterations in models of HPV infection to determine which of these events are important in the productive virus life cycle.

### 4.2. Lipid metabolism

Currently, there is very little understanding on how HR-HPVs alter lipid metabolism, and as a result, the available data come from large-scale studies which fall short of defining mechanisms. Analysis of data from TCGA revealed a higher proportion of upregulated genes involved in fatty acid β-oxidation in HPV-positive head and neck squamous cell carcinoma (HNSCC) samples compared to HPV-negative [57].

In a study comparing the plasma of patients with and without cervical cancer, genes involved in the biosynthesis of fatty acids, fatty acid metabolism and degradation, and phospholipid metabolism, were all downregulated in the plasma of cervical cancer patients [63]. Similarly, in serum samples from cervical cancer patients, very low density lipoproteins were increased while carnitine, a metabolite in fatty acid oxidation, was decreased compared to patients with CIN and chronic cervicitis [64]. However, it is important to note that the HPV status (positivity/type) was not specifically determined in the cervical cancer samples in this study and the role of HPV in lipid metabolism changes was assumed. It is clear that there is a need to further understand the mechanisms that drive the alterations to lipid metabolism in host cells in HPV infection and malignancy, however, the data available suggests that it is probable that lipid metabolism pathways are likely to be altered; whether this is a driver of cancer progression remains to be determined.

## 5. Kaposi's sarcoma-associated herpesvirus

Kaposi's sarcoma-associated herpesvirus (KSHV), also known as human herpesvirus 8 (HHV8), has an estimated seroprevalence ranging from 0 to 90%, dependent on geographical location. KSHV can infect a variety of human cells including endothelial cells, B cells and epithelial cells. Kaposi's sarcoma (KS) is a cancer of endothelial cells and while KS often occurs in human immunodeficiency virus (HIV)-positive individuals, KS can also occur in transplant recipients, and although rare, endemic and classic KS can also occur [65]. As such, almost 50% of HIV-positive individuals develop KS [66]. KSHV can also cause primary effusion lymphoma (PEL), a form of B-cell lymphoma [65].

### 5.1. Central carbon metabolism

The effects of KSHV on host endothelial cell glucose metabolism have been well established. An increase in glucose uptake and lactic acid production, two markers of aerobic glycolysis, was demonstrated in hTERT-immortalized microvascular endothelial (TIME) cells following KSHV infection [67]. Furthermore, KSHV-infected TIME and primary human dermal microvascular endothelial cells (hDMVECs) had a higher rate of basal extracellular acidification and lower oxygen consumption rates, which measure glycolysis and oxidative phosphorylation respectively. Together, these data suggest that KSHV-infected cells favour aerobic glycolysis over oxidative phosphorylation [67]. Glycolysis is necessary for KSHV virion production in TIME cells and when lactate dehydrogenase, an enzyme that catalyses the conversion of pyruvate to lactic acid, is inhibited using oxamate, there is a significant decrease in the abundance of KSHV protein latency-associated nuclear antigen (LANA)-positive cells [68]. Furthermore, oxamate-mediated inhibition of glycolysis induced a decrease in expression of viral open reading frame (ORF) 45, ORF59 (both early genes), ORF26 and K8.1 (late genes) in latent KSHV infected inducible endothelial cells (termed iSLK), suggesting that glycolysis is required for expression of viral genes at different stages of replication [68].

Some of the mechanisms through which KSHV regulates glycolysis in endothelial cell infection have been elucidated. Under normal hypoxic conditions, HIF*α* is stabilized, resulting in activation of genes containing hypoxia response elements, including those involved in glycolysis. However, latently infected hDMVECs and TIME cells upregulate transcription and protein expression of HIF*α* under normal oxygen conditions, suggesting that this contributes to the increase in genes involved in glycolysis [69]. Furthermore, in human umbilical vein endothelial cells (HUVECs) it was shown that PKM2 regulates the increases seen to aerobic glycolysis, and knockdown of PKM2 decreases the concentration of lactate in cells infected with KSHV, suggesting a decrease in aerobic glycolysis [70]. In latent infection, KS-positive lymphatic endothelial cells (LECs) and LECs expressing KSHV miRNAs had lower basal oxygen consumption rates and lactate secretion than control cells, suggesting a decrease in oxidative phosphorylation. This was demonstrated in combination with an increase in the stability of HIF1*α* and in the expression of GLUT1, an enzyme involved in glucose uptake, suggesting an increase in aerobic glycolysis; the expression of viral miRNAs contributed to enhanced growth under hypoxic conditions and this is important for maintaining viral latency [71]. In B cells, hypoxia induces high glucose uptake and lactate release in KSHV-infected BJAB cells, an EBV-negative Burkitt-like lymphoma cell line, and RNA sequencing analysis revealed that these cells had upregulated expression of genes involved in glycolysis, and a downregulation of genes involved in the TCA cycle. KSHV-infected BJAB cells stabilize HIF1*α*, and the viral G protein-coupled receptor (vGPCR), a target of HIF1*α* is a major viral antigen involved in metabolic reprogramming; mutant KSHV lacking vGPCR was unable to induce metabolic changes in an hypoxic environment [72].

Some of the metabolic changes detailed so far not only contribute to survival of KSHV in endothelial and B cells, but also contribute to tumorigenesis. PKM2 controls vGPCR-induced vascular endothelial growth factor (VEGF) secretion and oncogenesis and knockdown of PKM2 by siRNA reduces cellular migration and invasion and the angiogenic potential of KSHV-infected HUVECs [70]. Treatment of LECs with exosomes from KSHV-infected LECs which contain KSHV miRNAs induced a reverse Warburg effect with key changes associated with aerobic glycolysis [73]; the microenvironment plays a key role in the development of cancer, therefore manipulation of the cells surrounding those which form a significant proportion of tumour volume is key for tumorigenesis. Immunohistochemical staining of KS lesions showed elevated expression of HIF1*α* and HIF2*α* in cells positive for LANA; while dual-labelling was not used, the authors believe localization of HIF1*α* and HIF2*α* overlapped with LANA, confirming KSHV infection [69]. Based on the role of HIF1*α* in regulating the expression of genes involved in glycolysis and in KSHV infection, it is possible that the increase in HIF1*α* observed in the KS sections is due to the tumour undergoing higher rates of aerobic glycolysis. PEL cell lines also display an elevated rate of aerobic glycolysis compared to primary B cell controls. Upon inhibition of phosphoinositide 3-kinase (PI3K), there was a decrease in glycolytic flux [74]. Overall, KSHV appears to manipulate glycolysis in early infection to contribute to virus persistence. These metabolic changes may also promote carcinogenesis and the survival of cancer cells.

### 5.2. Lipid metabolism

While glucose metabolism has been well studied in KSHV infection and KSHV-induced carcinogenesis, it has also been demonstrated that KSHV alters host cell lipid metabolism. FASN is an important enzyme which plays a key role in regulating fatty acid metabolism. Human microvascular endothelial cells (HMVECs) and long-term infected telomerase-immortalized human umbilical vein endothelial cells (TIVE-LTCs) infected with KSHV display an increase in FASN mRNA expression [75]. Mass spectrometry analysis of TIME cells following KSHV infection demonstrated a rapid upregulation of long chain fatty acids (LCFA) [76]. Increases in the quantities of the fatty acid precursors choline and phosphocholine were also observed, as well as a decrease in the products of phospholipid degradation, glycerophosphorylcholine and glycerol-3-phosphate. Inhibition of FASN, which in turn inhibits fatty acid synthesis, resulted in a significant increase in cell death in KSHV-infected TIME cells [76]. Overall, these findings show that KSHV infection drives the synthesis of LCFA which is important for the survival of latently infected cells. In addition to an increase in LCFA synthesis in KSHV infection, there is an increase in lipid droplets that contain higher levels of neutral lipids [76,77]. This increase in lipid droplets may arise as a store for the excess lipids synthesized [76]. In KSHV-infected HUVECs, triglyceride levels were significantly higher in lytic infection (3 days post infection) before becoming significantly lower than the control in latent infection (24 days post infection), while cholesteryl esters were significantly higher 24 days post infection compared to the control [77]. Peroxisomes play a key role in lipid metabolism and signalling within cells. In TIME cells latently infected with KSHV, using mass spectrometry for proteomics analysis, it was identified that there is a significant upregulation of peroxisome-associated proteins 48 h post infection, which was also demonstrated through an increase in the number of peroxisomes in TIME cells, DMVECs and LECs up to 96 h post infection. Furthermore, knockdown of two key peroxisome-associated proteins, acyl-CoA oxidase (ACOX) 1 and ATP-binding cassette (ABC) D3 resulted in cell death of KSHV-infected TIME cells [78], highlighting a key role of peroxisomes in KSHV infection and life cycle.

As previously mentioned, the expression of cholesteryl esters is increased in latent infection and it was shown that inhibition of cholesteryl esterification causes impairment of HUVEC neo-angiogenic activity [77], suggesting that the synthesis of cholesteryl esters plays an important role in the development of KS. However, RNA sequencing analysis of KS biopsies compared to healthy tissue revealed a significant decrease in several lipid metabolism pathways and genes, including FASN [79]. This suggests that while FASN and lipid synthesis are important in promoting survival of KSHV during infection, the metabolic profiles of KS may differ in patient samples. However, it should be noted that the majority of infection studies use cell lines, which are homogeneous compared to tumours, which may be the cause of the apparent discrepancy. On the contrary, in KSHV-infected PEL cell lines, higher levels of FASN expression and fatty acid synthesis compared to primary B cells was noted, and inhibition of fatty acid synthesis results in cell death in KSHV-positive PEL cells [74]. This was identified to occur through PI3K, and inhibition of PI3K was shown to significantly reduce FASN expression in PEL cells and an increase in acylcarnitines, indicating incomplete oxidation of fatty acids in KSHV-infected PEL cells. The decrease in fatty acid synthesis was shown to be closely linked to glucose metabolism, as inhibition of glycolysis using 2-deoxy-d-glucose (2DG) also resulted in a decrease in fatty acid synthesis [74], highlighting the importance of understanding the interplay between different metabolic pathways.

Overall, it is clear that KSHV can alter glucose metabolism to favour aerobic glycolysis over oxidative phosphorylation. This not only occurs in early infection to contribute to the survival of the virus, but these changes can also contribute to a phenotype which favours tumour development, as well as persisting in KSHV-positive cancers to promote the survival of cancer cells. However, the role of lipid metabolism is less clear. While an increase in fatty acid synthesis plays a key role in the survival of infected endothelial cells, this may not be the case in KS, where fatty acid synthesis is downregulated.

## 6. Merkel cell polyomavirus

Merkel cell polyomavirus (MCPyV) has a seroprevalence of 60–80% in adults and infection is considered asymptomatic [80]. While it was first suspected that polyomaviruses were aetiological agents of cancer in animals in 1953 [81], it was in 2008 when an unknown human polyomavirus was detected in Merkel cell carcinomas and subsequently, named Merkel cell polyomavirus [82]. MCPyV has been shown to be present in 70–80% of Merkel cell carcinomas (MCC) [83], and despite MCC being a rare cancer, the incidence is rising [84].

As MCPyV is a relatively recently discovered virus and MCC is a rare cancer, there is very little literature describing MCPyV-driven alterations to host cell metabolism. However, in lung fibroblasts expressing MCPyV small T antigen (ST), there was an increase in the transcription of genes involved in glycolysis, including HK2 and several monocarboxylate transporters, compared to GFP-expressing cells. Furthermore, there was an increase in aerobic glycolysis and no significant alterations to oxidative phosphorylation, as measured by extracellular acidification rate (ECAR) and oxygen consumption rate, respectively, compared to GFP-expressing fibroblasts. MCC cell lines were determined to have variable levels of ECAR. However, this was not directly compared to the ST-expressing fibroblasts or MCPyV-negative MCC in this study and therefore the effect on MCPyV in MCC cannot be properly determined or compared to early infection [85]. While this is currently the only study which highlights that MCPyV ST can alter host metabolism, the authors briefly mention that their analysis revealed alterations to other metabolic pathways including downregulation of host genes involved in fatty acid oxidation, lipid metabolism and mitochondrial respiration, highlighting the potential for investigation in these areas.

## 7. Conclusion

**Figure 1.**
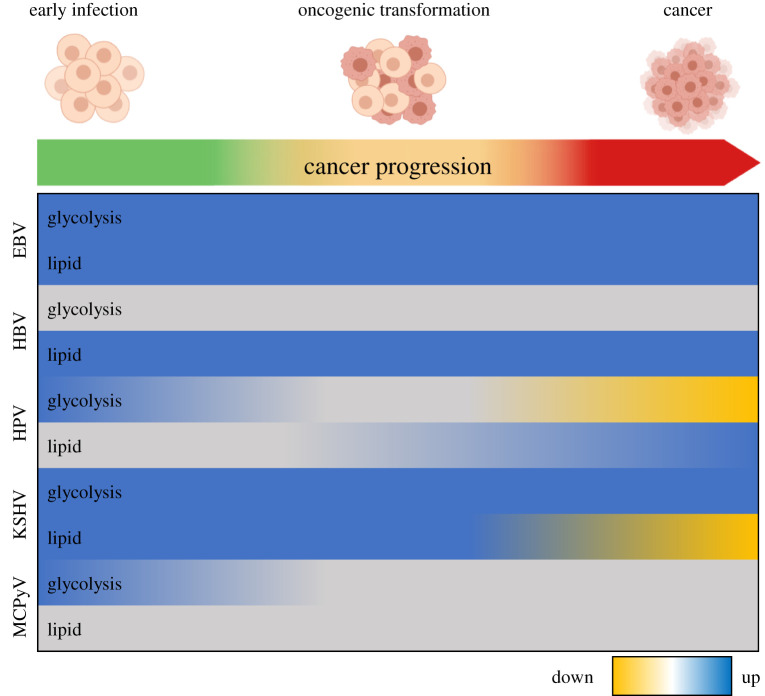
An overview of oncogenic DNA virus-induced changes to host glycolytic and lipid metabolism from infection to virus-driven cancer. Yellow shading indicates an overall downregulation of the metabolic pathway, while blue indicates a general upregulation. In the case of glycolysis, downregulation suggests an increase in oxidative phosphorylation. Grey represents cases where the direction of alteration is unknown.

This review has highlighted the various ways in which oncogenic DNA tumour viruses manipulate central carbon and lipid metabolism in early infection, oncogenic transformation and in virus-driven cancers (summarized in figure 1). In summary, EBV promotes a glycolytic phenotype combined with upregulation of lipid metabolism, specifically lipid synthesis, which contributes to oncogenic transformation and is maintained in EBV-driven cancers of both B cell and epithelial origin. By contrast, it is currently unclear whether there is a similar alteration in central carbon metabolism in early HBV infection. However, HBV appears to increase lipid synthesis and accumulation, which is consistent in HBV-driven cancer. Interestingly, glycolysis appears to be upregulated in HPV infection; however, in HPV-driven cancers, oxidative phosphorylation appears to be favoured over glycolysis, suggesting a shift in cellular energetics in HPV-positive cancers. The conclusions for lipid metabolism are less clear although some studies suggest that lipid metabolism is also altered by HPV. In KSHV, glycolysis is upregulated in early infection and in KSHV-associated malignancies, as well as promoting phenotypic changes involved in cancer development. Several lipid metabolic-associated pathways are increased in early KSHV infection and contribute to the development of a malignant phenotype, although these changes do not appear to persist in KSHV-associated cancers. While there is limited literature defining the manipulation of hose cell metabolism by MCPyV, it is highly likely that MCPyV alters host metabolism in a similar manner to other DNA tumour viruses.

Virus-induced host metabolic changes are clearly important in productive and/or persistent viral infection although in many cases the specific mechanisms involved are not well defined. The study of physiologically relevant models of viral infection will enable a more accurate understanding of how oncogenic DNA viruses hijack the cellular metabolic machinery. While there has predominantly been a focus on central carbon metabolism and glycolysis, investigation into other metabolic pathways has begun; as metabolic pathways are often interlinked, broadening the investigation of viral manipulation of host metabolism will allow for a clearer understanding of the global manipulation of metabolism by viruses. Further investigation could also provide novel targets for antivirals or anti-cancer agents; altering the source of fuel for tumours growth or virus replication is likely to be of therapeutic benefit. The increasing interest in the field of viral metabolism over the last decade has provided us with a greater insight into the changes to host molecular machinery, and it is proving to be an exciting and rapidly evolving field.

## Data accessibility

This article has no additional data.
